# *Mycobacterium* infection secondary to exogenous lipoid pneumonia caused by nasal drops: a case report and literature review

**DOI:** 10.1186/s12890-022-02265-8

**Published:** 2023-01-31

**Authors:** Huihong Wang, Shan Lu, Hequan Li, Yuehong Wang

**Affiliations:** 1 Department of Respiratory Diseases, The First Affiliated Hospital, Zhejiang University School of Medicine, Hangzhou, 310003 China; 2 Department of Respiratory Diseases, Taizhou Hospital of Zhejiang Province Affiliated to Wenzhou Medical University, Linhai, 317000 Zhejiang Province China

**Keywords:** Case reports, Mycobacterium, *Mycobacterium abscessus*, Nasal absorption, Nontuberculous mycobacteria, Pneumonia, Lipoid

## Abstract

**Background:**

Exogenous lipoid pneumonia (ELP) is a rare disease and its diagnosis is often mistaken or delayed. Secondary infection with rapidly growing non-tuberculous mycobacteria is a rare complication of lipoid pneumonia.

**Case presentation:**

A 38-year-old man presented with fever, cough, sputum, chest tightness, and shortness of breath. He had a 2-year history of allergic rhinitis and used liquid paraffin-containing menthol nasal drops daily. A chest CT scan showed multiple patchy ground glass opacities with blurred borders in both lungs, which were located in the inner pulmonary field and distributed along the bronchi. His ambient air PO_2_ was 63 mmHg. The patient was diagnosed with ELP by CT-guided lung biopsy. The nasal drops were discontinued, and systemic glucocorticoids were administered. During treatment, the pulmonary lesions deteriorated, and bronchoalveolar lavage was performed during bronchoscopy. Additionally, *Mycobacterium abscessus* was detected in the lavage fluid. Upon detection of a secondary *M. abscessus* infection, glucocorticoids were gradually discontinued, and anti-*M. abscessus* treatment was implemented. The patient’s symptoms rapidly ameliorated. After 11 months of anti-*M. abscessus* treatment, a repeat CT scan showed clear regression of the lung lesions.

**Conclusion:**

Routine microbiological examination of samples, including sputum or alveolar lavage fluid, is necessary for patients with diagnosed or suspected ELP.

## Background

Exogenous lipoid pneumonia (ELP) is caused by aspiration of oily substances into the lungs, and results in symptoms, such as acute and chronic inflammation of the lungs, local pulmonary fibrosis, and granuloma, which can affect gas exchange and lead to respiratory failure, or even death in severe cases [1]. Although such pathological features are helpful for the diagnosis of the disease, respiratory symptoms and imaging findings lack specificity. Furthermore, given its rarity, many clinicians are not familiar with ELP, which results in a high rate of misdiagnosis.

In patients with ELP with poor response to treatment, the possibility of a secondary infection with other microorganisms should be considered. Moreover, secondary infections with rapidly growing non-tuberculous mycobacteria (NTM) are rare complications of lipoid pneumonia [2, 3]. Here, we present a retrospective analysis of the diagnosis and treatment of a patient with *Mycobacterium abscessus* infection secondary to ELP caused by long-term application of liquid paraffin-containing menthol nasal drops, as well as a literature review on the topic.

## Case presentation

**Fig. 1:**
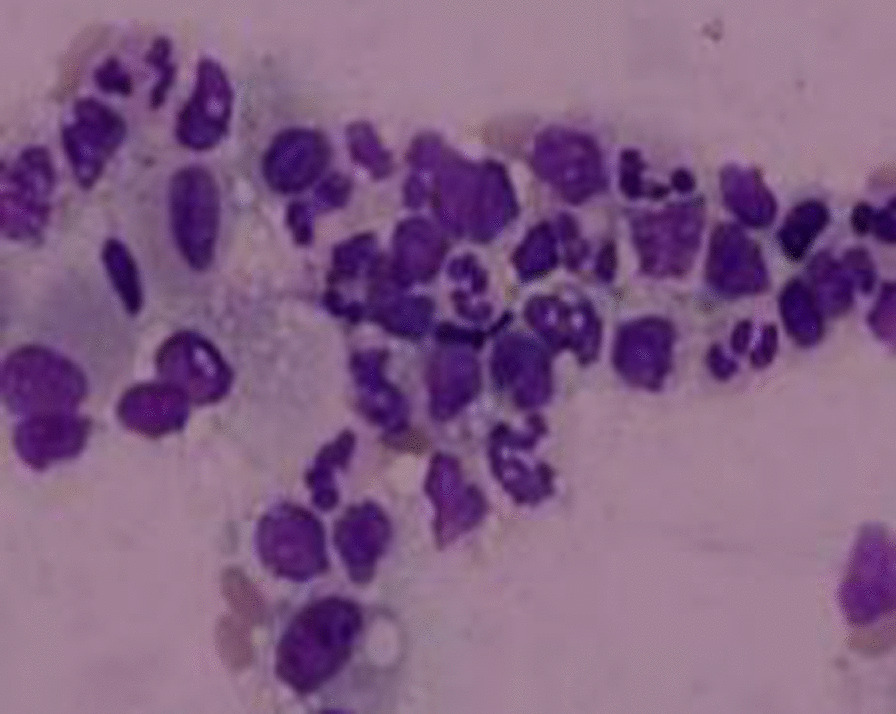
Cytological analysis of alveolar lavage fluid. The cytological analysis of alveolar lavage fluid shows a relatively large number of phagocytes and extracellular lipid droplets (H&E, ×400 magnification)

**Fig. 2:**
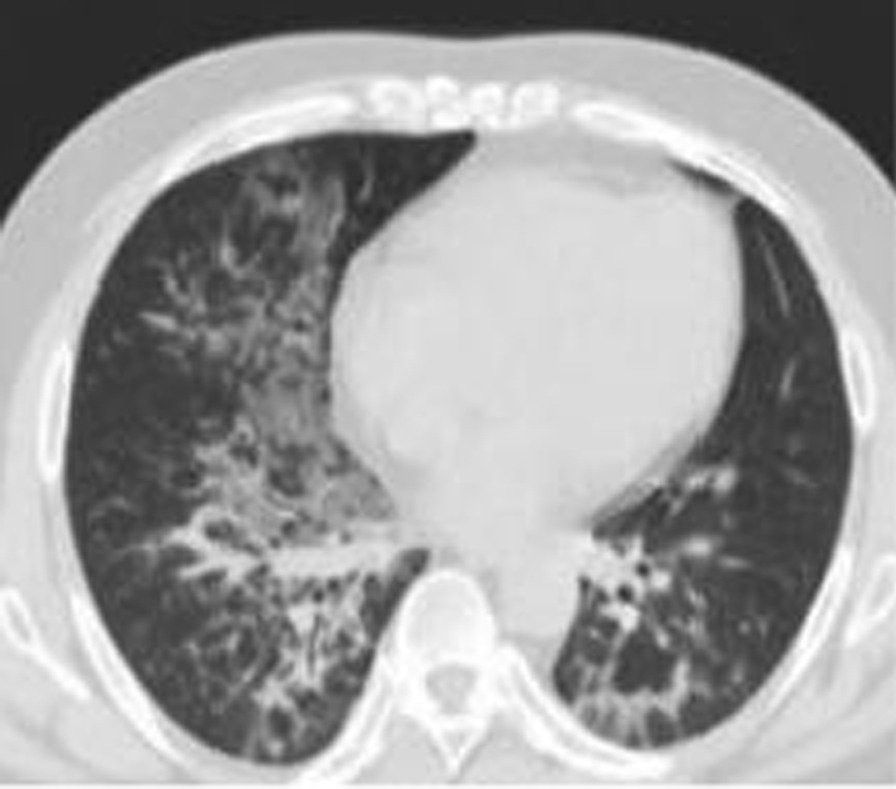
2020–5-30 chest CT scan revealing patchy ground glass opacities in the lungs. Patchy ground glass opacities are mainly located in the inner field, and distributed along the bronchi

A 38-year-old male with a history of chronic rhinitis who required the use of vigorously inhaled compound menthol nasal drops for more than 2 years, 5–20 times a day, in the supine position, was admitted for one day on 31 May, 2020 with complaints of fever, cough, sputum, chest tightness, and shortness of breath. The patient owned a Chinese toiletries store. He had a maximum body temperature of 38.9 °C with yellow purulent sputum, accompanied by white frothy sputum, muscle soreness in all four extremities, but no haemoptysis or skin rash. He had a body temperature of 37.0 ℃, a pulse rate of 101 beats per minute, a respiratory rate of 24 breaths per minute, and a blood pressure of 141/98 mmHg, with no obvious positive signs on cardiopulmonary auscultation on admission. Auxiliary examinations revealed a high-sensitivity C-reactive protein of 48.6 mg/L, a white blood cell count of 13.5 × 109/L, and an absolute neutrophil count of 10.1 × 109/L. HIV antibody was negative. A chest CT scan showed multiple patchy ground glass opacities in both lungs, with blurred borders, mainly located in the inner field and distributed along the bronchi (Fig. 1). A blood gas analysis showed a PO_2_ of 63 mmHg on room air. Cytological analysis of the alveolar lavage fluid under a bronchoscope showed 54% phagocytes, 32% neutrophils, 11% lymphocytes, and extracellular lipid droplets (Fig. 2). Gram-positive cocci were detected in alveolar lavage fluid smears. The bacterial culture and identification, fungal culture and identification, galactomannan detection test, fungal D-glucan assay, identification of Mycobacterium species (gene chip array) and acid-fast bacilli smear of the alveolar lavage fluid were all negative. The patient was diagnosed with community-acquired pneumonia, treated with 4.5 g of piperacillin sodium and tazobactam sodium IV every 8 h for nine days. A blood gas analysis showed a PO_2_ of 92 mmHg on room air on 5 June, 2020. Previous complaints of fever, chest tightness, and shortness of breath disappeared. The symptoms of cough and sputum improved. Hence, the patient was discharged from the hospital on 9 June, 2020. The patient developed fever again on 19 June, 2020, with a maximum body temperature of 40 °C, accompanied by chest tightness, cough, sputum and shortness of breath. Auxiliary examinations revealed a high-sensitivity C-reactive protein of 48.6 mg/L, a white blood cell count of 8.9 × 109/L, and an absolute neutrophil count of 6.4 × 109/L. A repeated chest CT scan showed consolidation of the lesions in the right lower lobe and the progression of scattered patchy opacities in both lungs. The patient was treated with 4.5 g of piperacillin sodium and tazobactam sodium IV twice a day for four days in the outpatient clinic, and admitted to hospital on 23 June, 2020 because the symptoms did not improve. Blood gas analysis showed a PO_2_ of 79 mmHg on room air. High-sensitivity C-reactive protein showed 48.6 mg/L, white blood cell count showed 8.9 × 109/L, and absolute neutrophil count showed 6.4 × 109/L. The patient was treated with 1 g of meropenem IV every 8 h and 600 mg of linezolid IV every 12 h, combined with 200 mg of voriconazole PO every 12 h from 23 June, 2020, for 5 days. A CT-guided biopsy of the mass in the right lower lobe was performed on 24 June, 2020, and pathological assessment revealed granulomatous inflammation. Previous complaints of fever, cough, sputum and chest tightness improved, but shortness of breath was aggravated. On 27 June, 2020, a high-sensitivity C-reactive protein analysis showed 15.2 mg/L, white blood cell count showed 7.7 × 109/L, and absolute neutrophil count showed 4.8 × 109/L. A repeated blood gas analysis on June, 2020 showed a PO_2_ of 65 mmHg on room air.

**Fig. 3:**
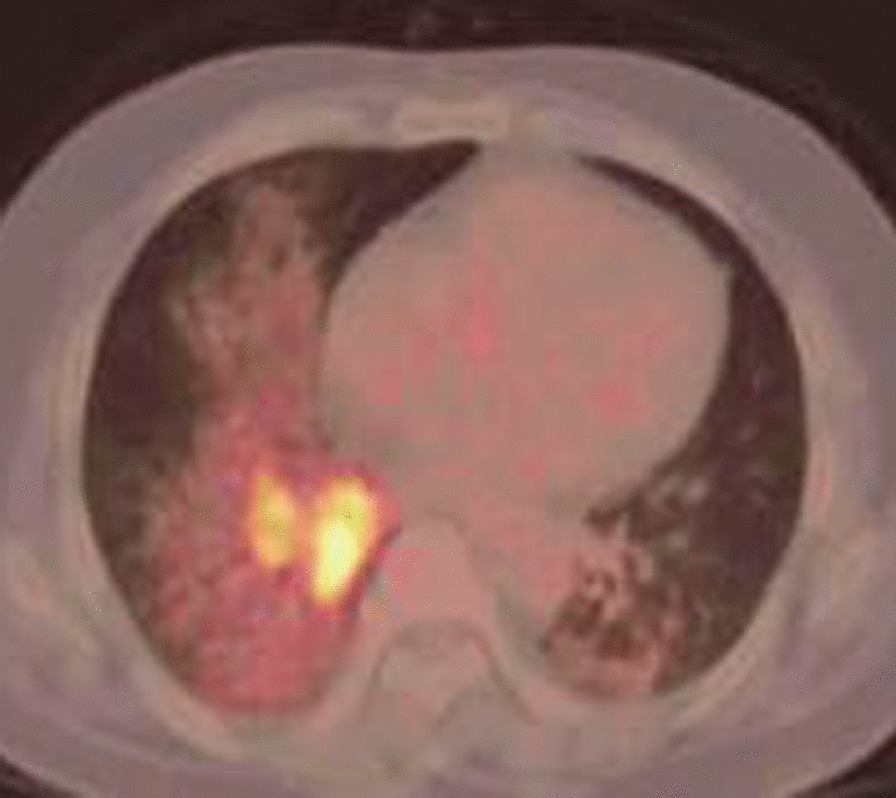
PET-CT scan showing patchy consolidation in the basal segment of the right lower lobe. PET-CT scan shows FDG metabolism was abnormally increased. FDG, fludeoxyglucose

**Fig. 4:**
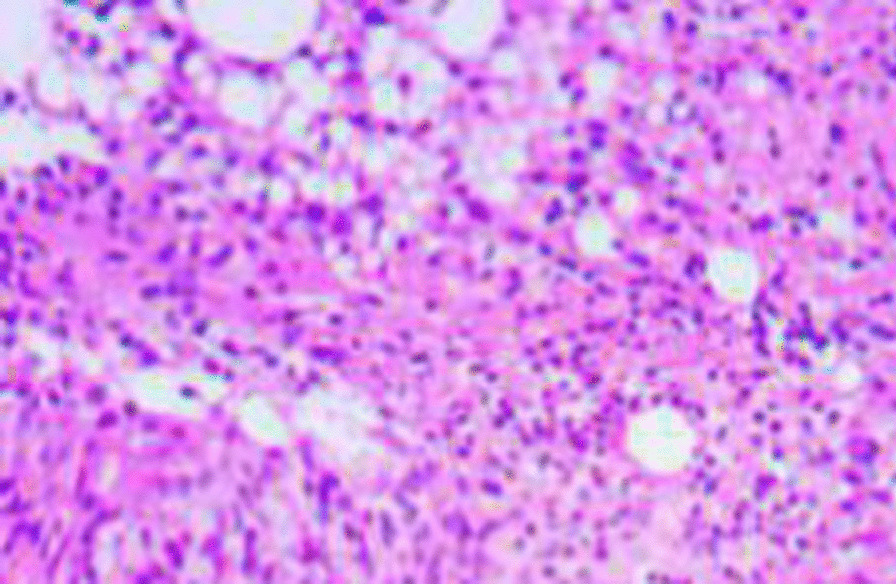
Haematoxylin and eosin stain of the lung biopsy specimen. The lung biopsy specimen from the nodular area of the right lower lobe shows epithelioid cell proliferation with numerous lipid droplets (H&E, ×50 magnification)

**Fig. 5:**
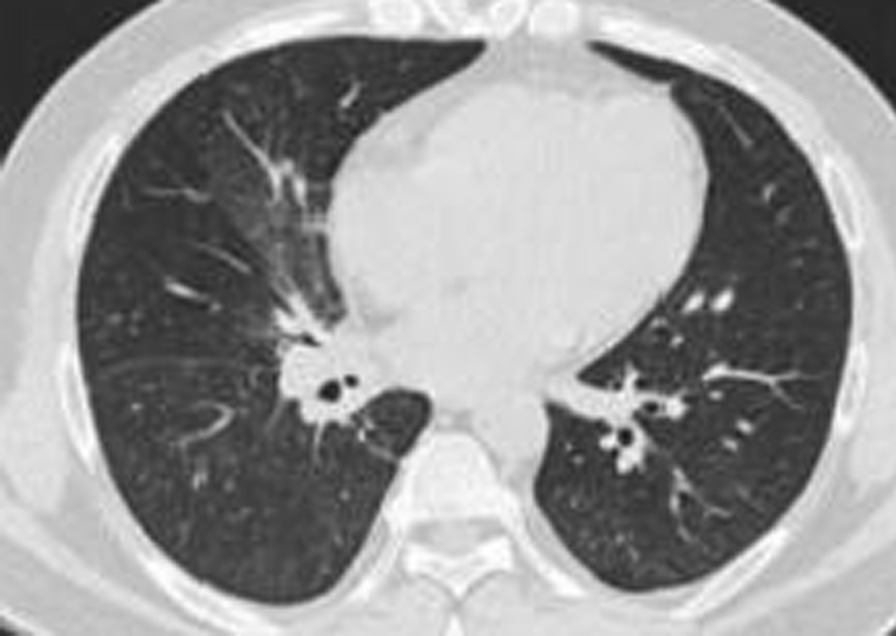
2021-7-27 repeated chest CT scan revealing bilateral lung lesions. A higher level of absorption was shown compared to previous scans

Therefore, he was transferred to the First Affiliated Hospital, Zhejiang University, on 29 June, 2020, and treated with cefoperazone sodium and sulbactam sodium IV every 8 h. A PET-CT scan was performed and showed patchy consolidations in the basal segment of the right lower lobe with abnormally increased fludeoxyglucose (FDG) metabolism; malignant lesions were, therefore, not excluded (Fig. 3). Pathology slides of the CT-guided biopsy, prepared in the Taizhou Hospital of Zhejiang Province by the First Affiliated Hospital, Zhejiang University, revealed chronic granulomatous inflammation (right lung biopsy) with a large amount of lipids, which were considered to be lipoid granuloma (Fig. 4). Therefore, he was diagnosed with ELP. On 2 July, 2020, administration of 40 mg of methylprednisolone IV once daily started, the compound menthol nasal drops, cefoperazone sodium and sulbactam sodium were discontinued. A repeat blood gas analysis on 5 July, 2020 showed a PO_2_ of 74 mmHg on room air. The patient was discharged on 9 July, 2020, after his symptoms improved, and was treated with 12 mg of methylprednisolone PO every 12 h. The dosage of methylprednisolone was tapered to one tablet every 2 weeks. During this period, a repeated chest CT scan revealed no obvious improvement of lung lesions on 3 August, 2020. The patient developed cough, sputum, fever with a maximum body temperature of 38 °C on 19 August, 2020, and a chest CT scan revealed an enlarged consolidation in the right lower lobe. High-sensitivity C-reactive protein showed 31.4 mg/L, white blood cell count showed 10.6 × 109/L, and absolute neutrophil count showed 8.4 × 109/L. Bronchoalveolar lavage fluid smears were positive for acid-fast bacilli 2+, and a mycobacterial DNA assay of the sputum identified *Mycobacterium abscessus*. The patient was diagnosed with pulmonary nontuberculous mycobacteriosis secondary to exogenous lipoid pneumonia. He was treated with 0.25 g of azithromycin PO once a day, 0.1 g of doxycycline PO every 12 h, for 14 months and 1.0 g of amikacin IV once a day, 2.0 g of cefmetazole sodium IV every 8 h, for 2.5 months from 26 August, 2020, and the dosage of methylprednisolone was gradually tapered off. The patient's temperature returned to normal and the cough and expectoration gradually resolved. Mycobacterium sputum culture, sent for examination on 19 November, 2020, was negative. A repeated chest CT scan on 27 July, 2021, revealed an obvious amelioration of the lung lesions (Fig. 5).

## Discussion and conclusions

Lipoid pneumonia is a rare type of pneumonia and can be classified in ELP and endogenous lipoid pneumonia. Endogenous lipoid pneumonia, also known as “cholesterol pneumonia” or “golden pneumonia,” is characterized by the production of lipids by the lung tissue itself and can be found in undifferentiated connective tissue disease, pulmonary alveolar proteinosis, primary sclerosing cholangitis, and other diseases [4]. Common risk factors for ELP include advanced or young age, abnormal anatomy or structure of the pharynx and oesophagus, such as Zenker’s diverticulum, gastroesophageal fistula, hiatal hernia, gastroesophageal reflux, achalasia, mental disorders, episodes of loss of consciousness, and neuromuscular diseases affecting swallowing or tract reflexes [5, 6]. In adults, the most common cause is the use of oily laxatives, such as olive oil, cod-liver oil, and liquid paraffin, to treat constipation, followed by the use of oil-containing nasal drops for chronic rhinitis [7]. The patient reported on in this article had a history of chronic rhinitis for 2 years with long-term use of large doses of compound menthol nasal drops in the supine position, each time by means of forceful aspirations. Meanwhile, oily substances, including mineral oil, animal oil, and vegetable oil, generally inhibit normal protective coughs and can impair the movement of cilia and hinder the expulsion of inhaled oily substances [8]. Over time, lipids accumulate in the lungs, which are engulfed by alveolar macrophages, thereby initiating local inflammatory responses, and causing damage to lung tissues [9].

The clinical manifestations of ELP are atypical and similar to those of other types of pneumonia, making it easy to misdiagnose. Additionally, they are a function of the type and amount of inhaled oily substance [10]. Betancourt et al. [11] reported that ELP could be divided into acute and chronic forms. Acute ELP is relatively rare, and usually occurs in pyrophiles or children. It is caused by aspiration of large amounts of oily substances in a short period, manifesting symptoms similar to acute respiratory infections, such as fever, cough, and dyspnoea. Lung imaging showed diffuse alveolar exudation. In contrast, chronic ELP is mainly caused by long-term, repeated aspiration of oily substances, and often occurs in the elderly or infants. It is characterized by chronic cough and sputum with occasional chest pain, and usually no fever. Some patients may be asymptomatic and present focal pneumonia with fibrosis, atelectasis, or tumour-like consolidation on lung imaging during routine physical examination [12]. Although the patient in this article had repeatedly inhaled menthol nasal drops over a long period, his clinical symptoms were acute, including cough, sputum, fever, chest tightness, and shortness of breath, with increased white blood cell count, elevated C-reactive protein, a decreased oxygenation index, and diffuse exudation on lung imaging. This may be related to the long-term aspiration of large amounts of nasal drops. Furthermore, the decrease in the oxygenation index may be explained by the inhalation of low viscosity liquid paraffin into the bronchus, which rapidly diffuses over the mucosa, leading to the destruction of surfactants, reduced lung compliance, severe pulmonary inflammation, and interstitial oedema [13]. The patient’s chest CT scans evolved from an initial diffuse alveolar exudation to tumour-like consolidations, indicating a gradual change from acute to chronic phase. Due to the consolidation in the right lower lobe, the possibility of malignancy could not be excluded. Therefore, a PET-CT scan was performed, which showed abnormally increased FDG metabolism of the local lesion. Similar to the report by Talwar et al. [14], this could have easily been misdiagnosed as a pulmonary malignancy. The patient was thus diagnosed by means of a pathological examination of bronchoalveolar lavage fluid and CT-guided lung biopsy. Granulomatous inflammation, which reveals the accumulation of lipid carrying macrophages, is the identifying pathological feature of lipoid pneumonia [10]. This is particularly helpful in distinguishing between lipoid pneumonia granuloma and mycobacterial granuloma that presents as coagulative necrosis, usually positive for acid-fast staining and detectable tuberculosis DNA.

Secondary infection with rapidly growing NTM is a rare but well-recognized complication of lipoid pneumonia [2, 15, 16]. Lipoid pneumonia can be superinfected with a variety of microorganisms, including *Branhamella catarrhalis*, *Pseudomonas*, *Acinetobacter*, *Klebsiella*, respiratory viruses, and rapidly growing NTM [8]. The following key words were used to search the PubMed database between January 1950 and September 26, 2021: “lipoid pneumonia” OR “pneumonia, lipoid” AND “*Mycobacterium*” OR “mycobacteria” OR “non-tuberculous.” After reviewing the titles, abstracts, and most of the full texts, we identified 18 articles describing 20 adult patients with lipoid pneumonia complicated by a rapidly growing NTM infection. Detailed information of the 20 patients is shown in Table 1. We found that *M. fortuitum*, *M. chelonae*, *M. smegmatis*, and *M. abscessus* were relatively more common in patients with rapidly growing NTM infections. However, in pulmonary NTM infections that are not related to aspiration, the primary pathogenic microorganisms are *M. avium-intracellulare* and *M. kansasii* [25]. Animal experiments have shown that, compared with saline solution, the pathogenicity of mycobacteria is enhanced when the lipid solution is injected subcutaneously. The impaired phagocytosis and bactericidal capabilities of lipid-laden macrophages are considered to be mechanisms of enhanced pathogenicity of NTM in a lipid-rich environment. The association of lipoid pneumonia with rapidly growing NTM is recognized in animals as well as humans [3, 29]. Therefore, clinicians should conduct examinations of sputum specimens or bronchoalveolar lavage fluid, including mycobacterial, bacterial, and fungal cultures, as well as acid-fast stains, in all patients with lipoid pneumonia, especially those with no improvement in clinical symptoms or imaging after the discontinuation of exogenous oily substances.

**Table 1:** Literature review of cases of exogenous lipoid pneumonia complicated by *Mycobacterium* infection

Case	Refs	Year	Age/sex	Risk factors	Aspiration	*Mycobacterium* infection	Symptoms	Treatment	Outcome
1	[17]	1967	62/M	Unknown	Mineral oil	Group IV atypical mycobacteria	Chest pain, fever	Antibiotics	Died
2	[18]	1968	85/M	Unknown	Mineral oil	*M. fortuitum*	Chills, fever, cough, coma, general muscular rigidity	Antituberculosis medication	Died
3	[16]	1970	54/F	Cardiac achalasia, constipation	Liquid paraffin, food	*M. fortuitum*	Fever, dyspnoea	Antibiotics	Died
4	[19]	1978	72/F	Constipation	Mineral oil	*M. fortuitum*	Chills, fever, cough	BAL, antibiotics	Died
5	[19]	1978	67/M	Parkinson’s disease, constipation	Laxatives of an unknown type	A rapidly growing mycobacterium that could not be fully classified	Fever, cough, unresponsiveness, hypotension	Antibiotics, antihypotensive medication	Died
6	[20]	1981	64/F	Esophageal achalasia, constipation	Mineral oil	*M. fortuitum*	Chills, fever, cough, night sweats, severe dyspnoea at rest	Antibiotics, steroids	Clinical condition much improved; no change on radiological images
7	[21]	1983	18/F	Nasal antrostomy	Petrolatum lubricant	*M. chelonae*	Fever	Antibiotics, steroids	No change
8	[22]	1986	Unknown	Tracheostomy after laryngectomy for carcinoma	Gomenol oil	*M. smegmatis*	Unknown	Unknown	Unknown
9	[23]	1987	Unknown	Unknown	Unknown	*M. fortuitum*	Unknown	Unknown	Unknown
10	[24]	1994	Unknown	Unknown	Unknown	*M. smegmatis*	Unknown	Unknown	Unknown
11	[25]	1996	56/F	Constipation	Liquid paraffin	*M. fortuitum*	Chest pain, fever	BAL, antibiotics	Clinical condition much improved; no change on radiological images
12	[26]	1999	63/F	Esophageal achalasia	Unknown	*M. chelonae*	Fever, tachycardia, dyspnoea, anorexia	Antibiotics, BAL	Much improved
13	[26]	1999	47/M	Constipation, hiatal hernia with reflux	Mineral oil	*M. fortuitum*	Fever, cough	Antibiotics	Much improved
14	[27]	2000	Unknown	Unknown	Unknown	*M. chelonae*	Unknown	Unknown	Unknown
15	[28]	2001	46/F	Total gastrectomy with oesophagojejunostomy, nausea, vomiting	Unknown	*M. abscessus*	Cough, expectoration, dyspnoea, fever	BAL	Unknown
16	[29]	2003	64/M	Constipation, total gastrectomy	Mineral oil	*M. abscessus*	Fever, cough	Antibiotics, steroids, BAL	Died
17	[14]	2004	82/M	Constipation	Mineral oil	*M. chelonae*	Asymptomatic	Surgery	Very much improved
18	[30]	2017	54/M	Gastroesophageal surgery, chef	High levels of fat aerosols	*M. abscessus*	Cough, fever	Antibiotics	Very much improved
19	[31]	2018	59/M	Total gastrectomy for gastric cancer, esophageal reflux	Unknown	*M. fortuitum*	Intermittent cough, sputum	Antibiotics, camostat, mosapride	Very much improved
20	[10]	2019	65/M	Allergic rhinitis	Petroleum jelly	*M. chelonae*	Cough	BAL, antibiotics	Very much improved

In terms of treatment, aspiration of oily substances should be immediately discontinued in patients with both acute and chronic ELP. Glucocorticoids are generally used in critically ill patients, but there is currently no consistent opinion on their recommended dose and course of application [32]. Some scholars [12] believe that early bronchoalveolar lavage may reduce the continuous damage of oily substances to the alveoli, and that some patients with poor response to medical treatment can be considered for surgery. In recent years, most patients with NTM infection secondary to lipoid pneumonia [2, 14, 28, 30] were reported to have recovered, well after discontinuing lipid exposure and undergoing appropriate antibiotic treatment, with or without a combination of glucocorticoids and bronchoalveolar lavage.

In conclusion, ELP is an uncommon type of pneumonia without specific clinical manifestations and clinicians generally have limited knowledge of this condition. Therefore, its diagnosis is often mistaken or delayed. Detailed medical history is critical for the diagnosis of ELP, especially when patients present with a history of lipid exposure. Furthermore, discontinuation of lipid exposure is important for successful treatment. Glucocorticoids and bronchoalveolar lavage should be performed, as appropriate, according to the patient’s condition. When lesions do not recess or progress during treatment, superinfections with other microorganisms, especially with rapidly growing NTM, should be considered.

## Data Availability

All data and materials are provided in the manuscript.

## Abbreviations

ELP: Exogenous lipoid pneumonia
FDG: Fludeoxyglucose
NTM: Non-tuberculous mycobacteria
